# Performance Evaluation Model of Agricultural Enterprise Technology Innovation Based on GA-BP Neural Network

**DOI:** 10.1155/2022/7110502

**Published:** 2022-06-23

**Authors:** Jian Kang, Minjuan Zhao

**Affiliations:** ^1^College of Economics and Management, Northwest A&F University, Yangling, Shaanxi 712100, China; ^2^Center for Shaannan Eco-Economy Research, Ankang University, Ankang, Shaanxi 725000, China

## Abstract

With the continuous development of my country's economy and society, how to effectively evaluate the technical innovation performance of agricultural enterprises has become the focus of research. This paper firstly processes and analyzes the technical innovation performance data of agricultural enterprises, and then processes and converts the technical innovation performance data of agricultural enterprises; then, through the analysis of the technical innovation performance data of agricultural enterprises, the key characteristics of the technical innovation performance of agricultural enterprises are excavated. Finally, a performance evaluation model based on agricultural enterprise technology innovation is proposed, and the validity of the model is verified with examples.

## 1. Introduction

At present, more than 60% of agricultural invention patents and more than 80% of new agricultural product development in China are completed by agricultural enterprises. With the continuous deepening of social and economic development led by scientific and technological innovation in my country, how to evaluate the innovation performance and management level of different types of scientific and technological innovation entities has become a major management problem to be solved urgently in scientific research and innovation. Develop a scientific, fair, and practical performance appraisal system and build an information-based and data-based management platform, which is helpful for the functional orientation of the main body of agricultural scientific research and innovation, problem-oriented mining, and development decision-making. With the continuous deepening of social and economic development led by scientific and technological innovation in my country, how to evaluate the innovation performance and management level of different types of scientific and technological innovation entities has become a major management problem to be solved urgently in scientific research and innovation. Develop a scientific, fair, and practical performance appraisal system and build an information-based and data-based management platform, which is helpful for the functional orientation of agricultural scientific research and innovation subjects, problem-oriented mining, and development decision-making. Small and medium-sized enterprises are not only effective carriers to accelerate the transformation of scientific and technological achievements and realize technological innovation but also an important source of national economic growth. Therefore, how to objectively, scientifically, and effectively evaluate the performance evaluation of agricultural enterprises' technological innovation is of great significance for promoting the development of agricultural enterprises' technological innovation and improving their core competitiveness. The concept of “technological innovation” was first put forward by economist Schumpeter, who defined innovation as establishing a new production function [1]. Since the 1980s, many scholars at home and abroad have conducted extensive research on technological innovation. In 1985, Musser made a systematic and overall analysis of technological innovation [2]. Technological innovation is defined as technological innovation based on the novelty of its conception and its successful realization of meaningful discontinuous events as features [3]. Fu Jiaji believes that the technological innovation capability of an enterprise refers to the conditions and power of technological development and transformation that an enterprise exhibits in the process of technological innovation activities [4]. At present, there are many methods for evaluating the technological innovation capability of enterprises, among which the widely used methods include the Delphi method, AHP, principal component analysis, grey system evaluation, data envelopment analysis, and fuzzy comprehensive evaluation. [5]. However, their disadvantage is that the random factors in the evaluation have more influence on the evaluation results, and the evaluation results are easily affected by the subjective consciousness, experience, and knowledge limitations of the evaluators [6, 7]. Backpropagation neural network is currently the most widely researched and applied artificial neural network in various fields. It embodies the most essential part of artificial neural network theory and application [8]. The technological innovation capability of an enterprise is affected by many factors. These influences are not isolated. They are interrelated and restrict each other, forming a complex nonlinear system [9]. As an effective tool to solve nonlinear system problems, this paper establishes a GA-BP neural network evaluation model to comprehensively evaluate the technological innovation ability of small and medium-sized enterprises [10].

The goal of technological innovation performance evaluation is to use scientific evaluation methods to measure and evaluate the completion of the established goals of agricultural enterprises based on selected comprehensive indicators and to objectively evaluate an enterprise through the results and put forward constructive suggestions [11, 12]. The main evaluation methods are the balanced scorecard method, the analytic hierarchy process, and other linear regression analysis methods, which are quite mature. Different from general enterprise performance evaluation, in the evaluation of main financial indicators, technological innovation performance evaluation has certain particularity. Du [13] first pointed out that priority should be given to improving inventory turnover rate and accounts receivable turnover rate. The weights of indicators that fit with the characteristics of agriculture are adapted to the characteristics of agriculture. Literature [14] established a performance evaluation system of agricultural enterprise technology innovation about lean manufacturing with the help of the balanced scorecard method and proposed an indicator system of four dimensions: customer, shareholder, production, and continuity. Wu [8] constructed a complete set of indicators for agricultural performance evaluation using grounded theory and proposed a measurement system for agricultural performance. The human brain is a very complex network structure, that is to say, the human brain completes intelligence, emotions, and other advanced psychological activities through a complex network structure, which is the original source of neural networks [15, 16]. Neural network is a technology that abstracts and simulates the general features of the human brain or nature [17–19]. In recent years, neural networks have achieved remarkable results in the field of prediction and empirical research. Compared with traditional prediction models, BP neural network has unique advantages [20] and has been widely used in enterprise performance, financial risk, and bank risk assessment. Many scholars have carried out effective combination innovation of BP neural network and enterprise evaluation system and verified the validity of the evaluation system by using the neural network model. Zuo et al. [21] used the neural network model to evaluate the breakthrough innovation performance of enterprises and analyzed and concluded that the BP neural network has good generalization ability in performance evaluation. Also using the BP neural network, Li et al. [22] constructed and verified the evaluation system of my country's listed banks. Li et al. [23] conducted research on private enterprises in my country, established a performance evaluation index system including enterprise objectives, partnership, and internal process, and combined neural network and dynamic fuzzy method to demonstrate the evaluation system. Other scholars such as Du [13, 14] applied the BP neural network model to evaluate the application.

The technical innovation performance evaluation of agricultural enterprises is affected by many factors [9]. These influences are not isolated. They are interrelated and restrict each other to form a complex nonlinear system. Therefore, based on the BP neural network model and combined with the genetic algorithm, this paper proposes the genetic algorithm-backpropagation neural network to comprehensively evaluate the technological innovation ability of small and medium-sized enterprises [24, 25]. First, analyze and process the technical innovation performance information of agricultural enterprises, and then construct a performance-based evaluation index system for small and medium-sized enterprises' technological innovation capabilities. Considering the entire process of production and operation, it includes two categories of indicators: resource input capability and innovation capability. Finally, combining BP neural network with the genetic algorithm, a performance evaluation model of agricultural enterprise technological innovation based on the GA-BP neural network is proposed. The identification method of electricity stealing behavior of energy Internet users based on GA-BP neural network can effectively strengthen the evaluation of technological innovation performance of agricultural enterprises. The research is aimed at different types of agricultural enterprises, on the basis of determining the positioning of agricultural enterprises' technological innovation performance evaluation, focusing on scientific and technological innovation, technology promotion, platform construction, talent team building and management level, select targeted and representative indicators. Build an information-based and data-based evaluation management platform to achieve rational evaluation and scientific decision-making.

## 2. Data Analysis and Processing of Technical Innovation Performance Evaluation of Agricultural Enterprises

**Figure 1:**
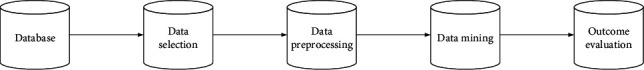
Data mining process.

Data mining refers to the science that extracts hidden laws from a large amount of data through systematic analysis and predicts the future or guides future work according to these laws. It is a process of extracting hidden and potentially useful information and knowledge [26, 27]. Data mining includes problem mining and raising, data processing, data mining analysis, and result evaluation. The process is shown in Figure 1.

1. *Problem Mining and Posing.* Through the analysis of the problems encountered in practical work and the corresponding data, the problem to be solved is proposed, and the appropriate mining algorithm is selected [28, 29]
2. *Data Processing.* Preprocessing the data according to the characteristics of the algorithm
3. *Data Mining Analysis.* Data mining algorithms are used to process data and dig out potentially valuable information

### 2.1. Data Processing of Technical Innovation Performance Evaluation of Agricultural Enterprises

In the technical innovation performance evaluation data, there are data missing caused by the acquisition. If these values are deleted, it will seriously affect the technical innovation performance evaluation and analysis results. Therefore, the missing part of the technical innovation performance evaluation data is filled with the Lagrangian interpolation method to ensure the technical innovation data enterprises. After realizing the frame-by-frame operation, the signal is windowed. In order to clearly show the effect, a rectangular window *w*(*n*) is selected. The calculation formula is

(1) $$\begin{array}{c} L_{n}\left(x\right)=\overset{n}{\underset{i=0}{\sum }}l_{i}\left(x\right)y_{i},\\ l_{i}\left(x\right)=\prod_{\begin{array}{c} j=0\\ j\neq i \end{array}}^{n}\frac{x-x_{j}}{x_{i}-x_{j}}, \end{array}$$

where *x* is the sequence number in the table below. *L*_*n*_(*x*) is the added interpolation result, and *x*_*i*_ is *y*_*i*_ the subscript sequence number of the original value.

### 2.2. Selection of Performance Evaluation Indicators for Technological Innovation of Agricultural Enterprises and System Construction

The “Performance Excellence Evaluation Criteria” was released in August 2004 by referring to the evaluation criteria of the American Markom Porich National Quality Award and combining the actual situation of my country's quality management. Starting from seven major modules, including the standard comprehensively, comprehensively, and systematically analyzes the entire process of enterprise operation, and all the factors involved to become an organization. Pursue standards of quality management and excellent performance. As a systematic and comprehensive evaluation criterion, the standard of excellent performance evaluates the quality management level of the enterprise and the overall performance of the enterprise. The overall level of an enterprise as a system includes not only the level of enterprise quality management and enterprise performance but also the operation level and innovation ability of the enterprise. As a reflection of the overall system level of the enterprise, the technological innovation capability of the enterprise can also be evaluated according to the performance excellence standard, and the entire process of enterprise operation can be analyzed and researched more comprehensively. The performance evaluation indicators of listed household appliance enterprises are listed in Table 1.

## 3. Identification Method of Electricity Stealing Users in Energy Internet Based on GA-BP Neural Network

**Figure 2:**
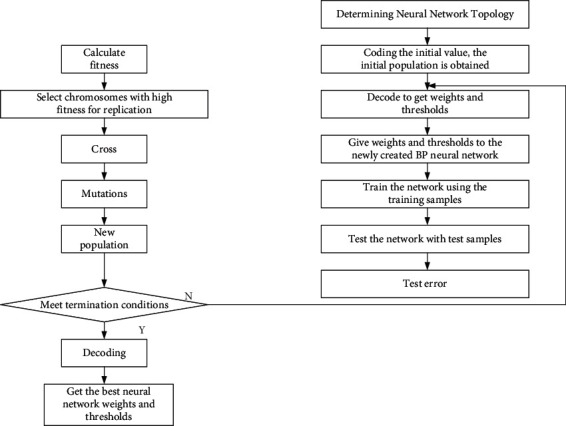
GA-BP network flow chart.

A genetic algorithm (GA) is a global search evolutionary algorithm that refers to the biological evolution process. Analogous to the biological evolution process, the genetic algorithm simulates biological evolution through selection, inferiority, and mutation to generate next-generation solutions. The heuristic optimal solution search method of the genetic algorithm combined with the BP neural network can effectively solve the problem that the BP neural network is easy to fall into the local optimal solution, and improve the accuracy of the technical innovation performance evaluation of agricultural enterprises. The specific GA-BP network process is shown in Figure 2.

The GA-BP neural network process is as follows.

### 3.1. Genetic Algorithm Initialization

Before using the genetic algorithm, it is necessary to decode the solution data in the solution space into genotype string structure data. The commonly used encoding method is the binary encoding method and different string structure data form different points. The N string-structured data generated by the encoding represent different individuals. Through the representation of different individuals, the birth population is finally formed, also known as the initial population.

### 3.2. Fitness Evaluation

Fitness indicates the individual's ability to adapt and also indicates the individual's pros and cons. The calculation formula of the individual's adaptability is

(2) $$\begin{array}{c} F=\text{mse}\left(Y-O\right)=\frac{1}{n}\sum_{i=1}^{n}{\left(y_{i}-o_{i}\right)}^{2},\\ F=\text{mse}\left(X-O\right)=\frac{1}{m}\sum_{i=1}^{m}{\left(x_{i}-|o_{i}\right)}^{2}, \end{array}$$

where *m* is the total number of samples; *x*_*i*_ is the output result of the genetic algorithm; *o*_*i*_ is the actual output result of the genetic algorithm, and mse represents the mean square error function.

### 3.3. Genetic Manipulation

The principle is applied to the population selection of genetic algorithm, and excellent individuals are selected for genetic reproduction of the next generation. Individuals with strong adaptability can pass good genes to offspring through inheritance. In this paper, the proportional selection strategy is adopted, the population data size is set as *M*, and then, the fitness formula of individual *i* is

(3) $$\begin{array}{c} p_{i}=\frac{k/F_{i}}{\sum_{i=1}^{N}k/F_{i}}, \end{array}$$

where *k* is the genetic coefficient.

In the genetic algorithm, the next generation of individuals is reproduced through the crossover operation, so as to obtain new individuals with new characteristics. The crossover operation is used to exchange genetic information and the mutant individual, which has the characteristics of small value.

### 3.4. Combination of Genetic Algorithm and BP Neural Network

**Figure 3:**
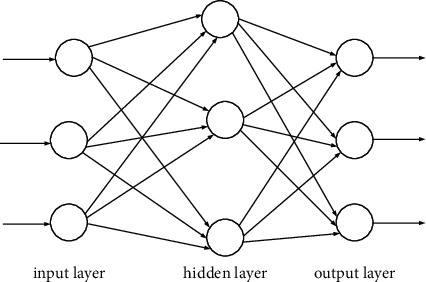
BP network topology.

Using the optimal value search of the genetic algorithm can reduce the calculation amount of the BP neural network and optimize the calculation process of the BP neural network. The fault tolerance of the entire network will be deteriorated, resulting in a reduction in the ability to recognize unlearned samples. For the irregular content in the sample, its generalization ability will also be reduced. Previous research theories have confirmed that for a typical 3-layer BP neural network, when the number of input layer units is *n* and the number of hidden layer units is 2*n *+ 1, the network can approximate any differentiable with arbitrary precision function. The BP neural network maps the input variables to the output variables nonlinearly through the excitation function and continuously adjusts the weight threshold of each layer connection. The BP neural network draws on the model structure of the neural network and builds a highly complex learning system through the interconnection of a large number of neurons. The hidden layer performs data calculation by setting one or more layers of neurons, and each layer of neurons can have several nodes. Double-hidden BP neural network is commonly used in a pattern classification problem, which has the advantage of fast classification speed, so the BP neural network structure with double hidden layer is adopted in this paper. The neural network is adjusted through the weight matrix and error feedback between the layers to achieve the expected output result of the load. Compared with the traditional artificial neural network, the double hidden layer BP neural network has improved in parallel processing of massive data and accuracy. BP network topology is shown in Figure 3.

The processed data enter the second step of processing, and the frame-by-frame operation is realized by using the method of field overlap. In order to accurately analyze the data, the data are divided into *t* frames, and the output of the first hidden layer node i is

(4) $$\begin{array}{c} b_{i}=f\left(\sum_{i=1}^{m}w_{\text{mi}}a_{m}-\theta_{i}\right). \end{array}$$

The output of the second hidden layer node *j* is

(5) $$\begin{array}{c} c_{j}=f\left(\sum_{j=1}^{I}w_{\text{ij}}b_{i}-\theta_{j}\right). \end{array}$$

The output result of the nth node of the output layer is

(6) $$\begin{array}{c} d_{n}=f\left(\sum_{n=1}^{J}w_{\text{jm}}c_{j}-\theta_{n}\right). \end{array}$$

In order to improve the convergence speed of the BP neural network, the input data are normalized to reduce the range of changes and improve the flexibility of interval selection. The formula is as follows:

(7) $$\begin{array}{c} t_{i}^{\prime }=\frac{t_{i}-t_{\min}}{t_{\max}-t_{\min}}, \end{array}$$

where *t*_*i*_ is the input value of the model, *t*_*i*_′ is the normalized value of the set [0, 1] interval, *t*_min_ and *t*_max_ are the upper and lower limits of the set value, respectively.

The activation function is as follows:

(8) $$\begin{array}{c} f\left(x\right)=\frac{1}{1+e^{-x}}. \end{array}$$

In view of the above analysis, through the collection and analysis of user electricity consumption information, a method for identifying electricity stealing behavior of energy Internet users has a very positive effect on the long-term safety.

The analysis process is described as follows:

1. Create a database. Analyze, collect, and process all agricultural enterprise technological innovation performance evaluation data during the period.
2. Analyze the GA-BP neural network in the database. Collect mining potential information of technical innovation performance evaluation of agricultural enterprises.
3. Develop a plan, i.e., plan for the performance evaluation of agricultural enterprises' technological innovation that has been excavated.
4. Compare information, i.e., compare the information in the forecast with the known information, and then deploy according to the previously formulated plan.

## 4. Experimental Analysis

At the beginning of the model iteration, the fitness value of the individual population is far from the optimal fitness, and the individual fitness value increases significantly. In the later stage of the model iteration, due to the continuous convergence of the model, the fitness value of the individual population is getting more and more close to optimal fitness. After completing the above work, the global optimal initial weight threshold value searched by the genetic algorithm can be obtained, and the initial weight threshold value is brought into the network to train the BP network. Set the training parameters of the BP network: the number of neurons in the input layer is 6, the number of neurons in the hidden layer is 9, and the number of neurons in the output layer is 1. Select the unipolar SigmoID function as the transmission. For the selection of function, the gradient descent BP training algorithm function training is selected as the training function, where the parameter is set as follows: the maximum number of training times is 200 times, the learning rate is 0.1, and the learning minimum mean square error target is 0.003. According to the selected 12 input vectors, the output data are normalized.

According to the constructed model, a BP neural network model with two hidden layers is established by using Matlab, and the normalized data are input to iterate until the output result is full and the prediction reaches the accuracy required by the experiment. Compared with a BP neural network, GA-BP neural network is closer to the technical innovation performance evaluation of agricultural enterprises.

**Figure 4:**
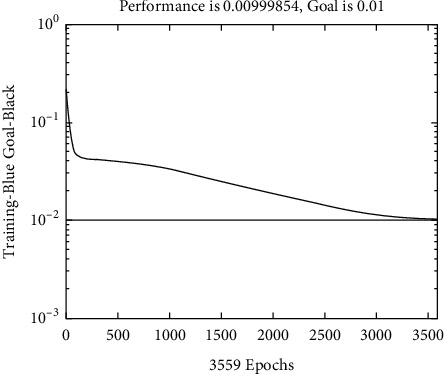
Experimental results.

The results of the experiment can be seen in Figure 4. The error between the predicted value and the actual value of the test sample is shown in Table 2.

By testing the test set composed of the technical innovation performance evaluation data of agricultural enterprises in 2020, the relative error between the test results and the expected results meets the requirements of the target error, which can effectively indicate that the constructed network model has very good performance. It can be seen that the evaluation results are very close to the actual results, which verifies the feasibility of the GA-BP neural network model in the performance evaluation of agricultural enterprises' technological innovation.

## 5. Conclusion

This paper proposes a model based on GA-BP neural network which provides a scientific method. The adopted GA-BP neural network algorithm can realize arbitrary linear and nonlinear function mapping, avoid artificial determination of weights, and reduce the evaluation of the evaluators in their cognition. Through systematic training and learning, the system error can meet the accuracy requirements. Research on the performance of agricultural enterprises can solve the problem of fairness evaluation between different types and different responsibilities. Analyzing and evaluating the differences in different aspects of the technological innovation performance of agricultural enterprises from different levels, to find out the gaps between the evaluation subjects, is conducive to development decision-making and development of targeted development plans. The whole set of evaluation index system proposed by the research is suitable for other nonprofit agricultural enterprises and has certain promotion and application value. For nonpublic scientific research institutes and technology enterprises, it is also possible to increase the relevant indicators such as industrial income or adjust the weight of the evaluation indicators on the basis of the evaluation system. The constructed performance evaluation database management system can realize the networkization of performance evaluation workflow management, evaluation index management, and reporting data management, and realize the whole process automation of performance evaluation, calculation and score, data statistics, and result release, and has system expansion functions. These high-quality functions are conducive to the promotion and application of the system. The algorithm overcomes the shortcomings of BP, improves the calculation speed and processing performance compared with the statistical algorithm and dynamic memory algorithm, is more suitable for the task of technological innovation performance evaluation, and provides a new idea for the field of technological innovation performance evaluation of agricultural enterprises.

## Figures and Tables

**Table 1:** Performance evaluation indicators of listed home appliance manufacturers.

First-level indicator	Secondary indicators	Three-level indicator
Ability to invest resources	Human resources	Number of scientific and technical personnel
		Structure of scientific and technical personnel
		The proportion of scientific and technological personnel in the total number of people
	Financial resources	Total investment in technological innovation
		Technological innovation investment structure
		The proportion of technological innovation investment in sales revenue
	Information resource	Information resource base construction
	Infrastructure	Number of experiments and test equipment
		Experiment and test equipment structure
	Related party relationship	Investment in the construction of supplier and strategic partnership
		Supplier and strategic partnership maintenance investment
Innovation process capability	Innovation value creation process	Identification of the innovation value creation process
		Determination of requirements for innovation value creation process
		Design of innovative value creation process
		Implementation and improvement of innovation value creation process
	Innovation support process	Identification of innovation support processes
		Determination of innovation support process requirements
		Design of the innovation support process
		Implementation and improvement of the innovation support process
Innovation output capability	Quality	The advanced nature of technical performance indicators of innovation achievements
		Customer satisfaction and loyalty for innovation outcomes
		Innovation outcome cost structure
		The difference between the cost of innovation and other similar products or substitutes degree
	Type	The average development cycle of innovation achievements
		Average time between production of innovations
	Difference	The structure of innovation achievements
		Performance results of innovation outcomes
		Market share of innovative achievements
	Finance	Operating income from innovation achievements
		Total profit of innovation achievements
		Contribution rate of total assets of innovation achievements
Innovation environment support	External environment	Contribution rate of government policies to enterprise innovation
		Total financial investment
		Total loans of financial institutions
		Contribution rate of public infrastructure to enterprise innovation
		Contribution rate of regional innovation level to enterprise innovation
		The level of competition in the regional market
	Internal environment	Leadership's innovative ideas and corporate innovation culture atmosphere
		The position and target planning of enterprise innovation in overall strategy
		Human resources work system, incentive mechanism and assessment

**Table 2:** The error between the predicted value and the actual value.

Serial number	26	27	28	29	30
Deviation	−0.084	0.009	−0.052	0.060	−0.006

## Data Availability

The dataset can be accessed upon request.
